# 

**Published:** 1857-10

**Authors:** 


					﻿g@=° Circumstances unavoidable and unnecessary to detail,
have prevented the editors from giving the usual attention to
the Journal, we hope to make amends in the future, asking
the indulgence of our friends to any apparent negligence in
this number.
jgsgr1 As faithful Journalists we record the claim of Prof. J.
Adams Allen to the discovery of the Excito-Secretory func-
tion. "Whatever may be the facts in reference to priority
upon this subject, Dr.’s Ilall, Campbell and Allen, are each
entitled to great credit for their investigations and discoveries
in Physiological science, and neither, perhaps the less, on ac-
count of the labors and contributions of the others.
TO CORRESPONDENTS.
All Communications pertaining to the Editorial Department of the Journal,
8hould be addressed to the Editors.
pgr- All Communications, having reference to the Subscription, the Advertising
or the Financial Department, should be addressed to G. P. Eddy & Co.
				

